# Effects of herpes zoster vaccination and antiviral treatment on the risk of stroke: a systematic review and meta-analysis

**DOI:** 10.3389/fneur.2023.1176920

**Published:** 2023-05-17

**Authors:** Yong-hui Jia, Yu-bo Dong, Hai-yin Jiang, Ai-juan Li

**Affiliations:** ^1^Pharmacy Department, The 960th Hospital of PLA, Jinan, China; ^2^State Key Laboratory for Diagnosis and Treatment of Infectious Diseases, Collaborative Innovation Center for Diagnosis and Treatment of Infectious Diseases, The First Affiliated Hospital, College of Medicine, Zhejiang University, Hangzhou, China

**Keywords:** infection, vaccine, cerebrovascular, virus, pathogen

## Abstract

**Background:**

Evidence suggests that there is an increased risk of stroke after herpes zoster (HZ). However, reports on the effects of HZ vaccination (HZV) and antiviral treatment on stroke risk are inconsistent. Thus, we examined these associations in a meta-analysis.

**Methods:**

To identify relevant studies, we searched three databases for articles published up to January 2023. Random-effect models were examined to determine overall pooled estimates and 95% confidence intervals (CIs).

**Results:**

This review included 12 observational studies (six on HZV and seven on antiviral treatment). When comparing vaccinated and unvaccinated patients, vaccination was found to be associated with a lower risk of stroke (OR, 0.78; 95% CI 0.68–0.9; *P* = 0.001). A meta-analysis of self-controlled case series (SCCS) revealed evidence of a reduced OR in individuals who received the vaccine (OR, 1.14; 95% CI 0.94–1.37; *P* = 0.181) compared with unvaccinated individuals (OR, 1.36; 95% CI 1.15–1.61; *P* < 0.001). Compared with untreated patients, antiviral therapy was not associated with a reduced risk of stroke (OR, 1.13; 95% CI 0.94–1.36; *P* = 0.201). The meta-analysis of the SCCS showed no evidence of a reduced OR in individuals who received antiviral therapy (OR, 1.33; 95% CI 1.17–1.51; *P* < 0.001) compared to untreated individuals (OR, 1.45; 95% CI 1.25–1.69; *P* < 0.001).

**Conclusions:**

This meta-analysis suggests that the HZV, but not antiviral treatment, decreases the odds of developing stroke.

## Introduction

Primary infection with varicella zoster virus (VZV) usually occurs in childhood, causing chickenpox characterized by a vesicular pruritic rash, viremia, and fever (1). VZV becomes latent in the dorsal root ganglia and nerve cells and can reactivate to cause herpes zoster (HZ), which typically manifests as localized, painful, dermatomal vesicles or blisters (2). The incidence of HZ has increased from 1.7 in 1993 to 7.2 per 1,000 person-years in 2016, with a substantial negative impact on the health-related quality of life (3). In the last decade, infectious diseases have been identified as a new risk factor for stroke. Several pathogens have been recognized as being directly associated with the development of stroke (4–6). Preclinical studies have demonstrated that VZV triggers a variety of inflammatory effects that may contribute to thrombogenesis, atherosclerosis, and vasculopathy and thus to an increased risk of stroke (7–9). Recent research on the effect of HZ on stroke has reported an increased risk of stroke or myocardial infarction in HZ patients, especially ophthalmic zoster patients (10, 11). Given the high morbidity and mortality of stroke, any potential prevention strategy becomes increasingly important.

With the introduction of the zoster vaccine live (ZVL, Zostavax) in 2006 and the recombinant zoster vaccine (RCV, Shingrix) in 2017, HZ vaccination (HZV) has reduced the risk of developing HZ and postherpetic neuralgia (12, 13). Some studies have examined the association between HZV and post-HZ stroke, but the findings have been inconsistent (14–19). One study had a within-person study design but did not find a protective role of HZV (14). The most recent case–control study conducted to date found a reduced risk of stroke in vaccinated patients compared with unvaccinated patients (19). Additional studies have explored whether antiviral treatment modifies the risk of stroke after HZ. Some studies have demonstrated that antiviral treatment following HZ is associated with a reduced risk of stroke (20, 21), while others have found no difference in stroke risk between patients treated (or not) with antivirals (16, 22–25). To the best of our knowledge, the latest published review on this topic included articles in PubMed up to January 2017, including only two observational studies evaluating the association between antiviral treatment and stroke. Furthermore, this study did not conduct a meta-analysis (26); hence, the researchers were unable to provide an overall quantitative summary of their results. The effect of HZV and antiviral treatment on post-HZ stroke remains unclear. Therefore, we conducted a systematic review and meta-analysis on this topic, including all observational studies published up to January 2023 to examine whether HZV and antiviral treatment modify the risk of post-HZ stroke.

## Methods

This meta-analysis is reported according to the Preferred Reporting Items for Systematic Reviews and Meta-Analysis (PRISMA) (27).

### Search strategy

This systematic review examined English-language publications in the EMBASE, PubMed, and Cochrane Library databases. The literature was searched using the following keywords from inception dates to 16 January 2023: “Herpes Zoster OR Shingles OR Zoster OR VZV OR zona” and “stroke OR cerebral arterial disease OR myocardial infarction OR ischemic attack OR cerebral ischemia.” The reference lists of identified publications and relevant reviews were also searched manually.

### Study selection

Peer-reviewed publications were considered eligible for data extraction if they were observational studies with cross-sectional, case–control, case–crossover, self-controlled case series (SCCS), or cohort study designs; compared vaccinated with unvaccinated patients and/or antivirally treated with untreated patients; reported odds ratios (ORs), relative risks (RRs), incidence ratios (IRs), and/or hazard ratios (HRs) of associations; and included adequate data to derive risk estimates. Reviews, case reports, conference abstracts, editorials, correspondence, basics, and animal studies were excluded.

### Data extraction and quality assessment

An Excel spreadsheet was used to record the details of the included studies, including the first author's name, year of publication, country, study design, study period, age, number of observation groups, information regarding HZ exposure, diagnostic criteria for stroke (stroke or myocardial infarction), outcome measures, and statistical adjustments.

Two authors evaluated study quality using the Newcastle–Ottawa Quality Assessment Scale (NOS), which was developed for assessing observational studies (28). This scale is divided into three domains: participant selection (four questions), study group comparability (two questions), and outcome (three questions), with all questions having a value of one. Studies with a quality score ≥7 were considered to be of high quality.

### Data analysis

Meta-analysis was performed using STATA version 10.0 (StataCorp, USA). Heterogeneity among the included studies was assessed using the *I*^2^ statistic (29). *I*^2^ values of 25%, 50%, and 75% represent low, medium, and high heterogeneity, respectively (29). Due to differences in the study populations and methodology, effect estimates were combined using the random-effects generic inverse variance method of DerSimonian and Laird (30). A pooled OR was calculated. Publication bias was not assessed because fewer than 10 studies were included in the meta-analysis (31). Statistical significance for all analyses was set at a *P-*value of <0.05.

## Results

### Search results

**Figure 1:**
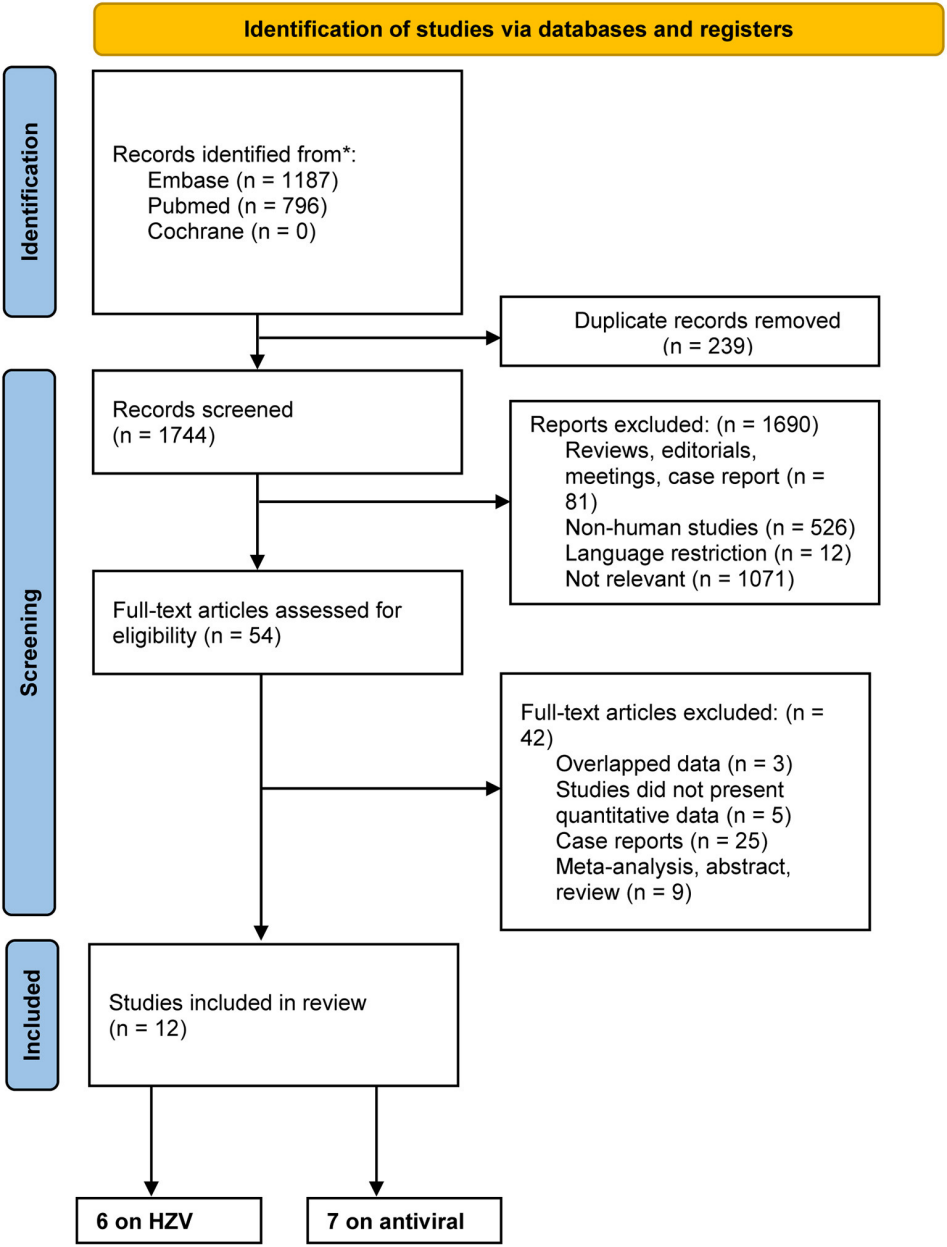
Flowchart of the search process and study selection.

Using the keywords, our comprehensive search identified 1,744 articles after excluding 239 duplicates, of which 996 were excluded after the initial screening of titles and abstracts. Then, the full text of 54 articles was reviewed to determine their eligibility. As a result, our analysis included 12 studies [six (14–19) on HZV and seven (16, 20–25) on antiviral exposure]. The screening and reason for exclusion at each step are shown in the PRISMA flow diagram (Figure 1).

### Characteristics of the included studies investigating the association between HZV and stroke

Table 1 shows the characteristics of the six studies that investigated the association between HZV and stroke risk; there were three (14–16) SCCS studies, two (17, 18) cohort studies, and one (19) case–control study. Publication years ranged from 2015 to 2022, and five studies were conducted in the United States. Only one (18) was a hospital-based study; the others were population-based studies that analyzed database data. Participants in four studies (14–17) were older than 65 years old. Four studies (14–17) evaluated only ZVL (Zostavax), one study (18) evaluated only RZV (Shingrix), and the remaining study (19) evaluated both ZVL and RZV. Based on the NOS quality assessment scores, all studies were deemed to be of high quality. The score breakdown is presented in Supplementary Tables S1, S2.

**Table 1:** Characteristics of the included studies investigating the association between HZV and cerebrovascular events.

**References, study location**	**Study design**	**Study period**	**Age (y)**	**Comparison**	**Number of events**	**Definition of stroke**	**Ascertainment of HZV exposure**	**Type of HZV**	**Adjustment**	**Quality**
Minassian et al. (14), USA	SCCS	2006–2011	≥65	Pre-vaccination vs. after-vaccination	42,954	ICD-9-CM	Medicare part D drug files	ZVL	Age	9
Totterdell et al. (15), Australia	SCCS	2016–2018	70–79	Pre-vaccination vs. after-vaccination	2,166	Medicine insight data	Medicine insight	ZVL	Age	8
Yang et al. (16), USA	SCCS	2008–2017	≥65	Pre-vaccination vs. after-vaccination	87,405	ICD-9-CM or ICD-10-CM	Medicare provider analysis and review	ZVL	FDR adjusted	9
Yang et al. (17), USA	Cohort	2008–2017	≥65	Vaccinated vs. unvaccinated	50,681	ICD-9-CM or ICD-10-CM	Medicare provider analysis and review	ZVL	Propensity score matching	9
Nelson et al. (18), USA	Cohort	2018–2019	≥50	Vaccinated vs. unvaccinated	308	ICD-10-CM	Electronic health record	RZV	Age, gender, study site, a dermatology visit, an optometry visit, and prior zoster vaccine live vaccination, hypertension, diabetes, hyperlipidemia, and ischemic conditions	9
Parameswaran et al. (19), USA	Case-control	2010–2020	≥18	HZ vs. no HZ	14,523	ICD-9 or ICD-10	Veterans affairs corporate data	ZVL or RZV	Age, gender, zoster history, congestive heart failure, diabetes, renal failure, cancer, peripheral vascular disease, paralysis, COPD, HIV/AIDS,/metastatic cancer, myocardial infarction, CVA, dementia, rheumatologic disease, and liver disease	8

### Meta-analysis of HZV and risk of stroke

**Figure 2:**
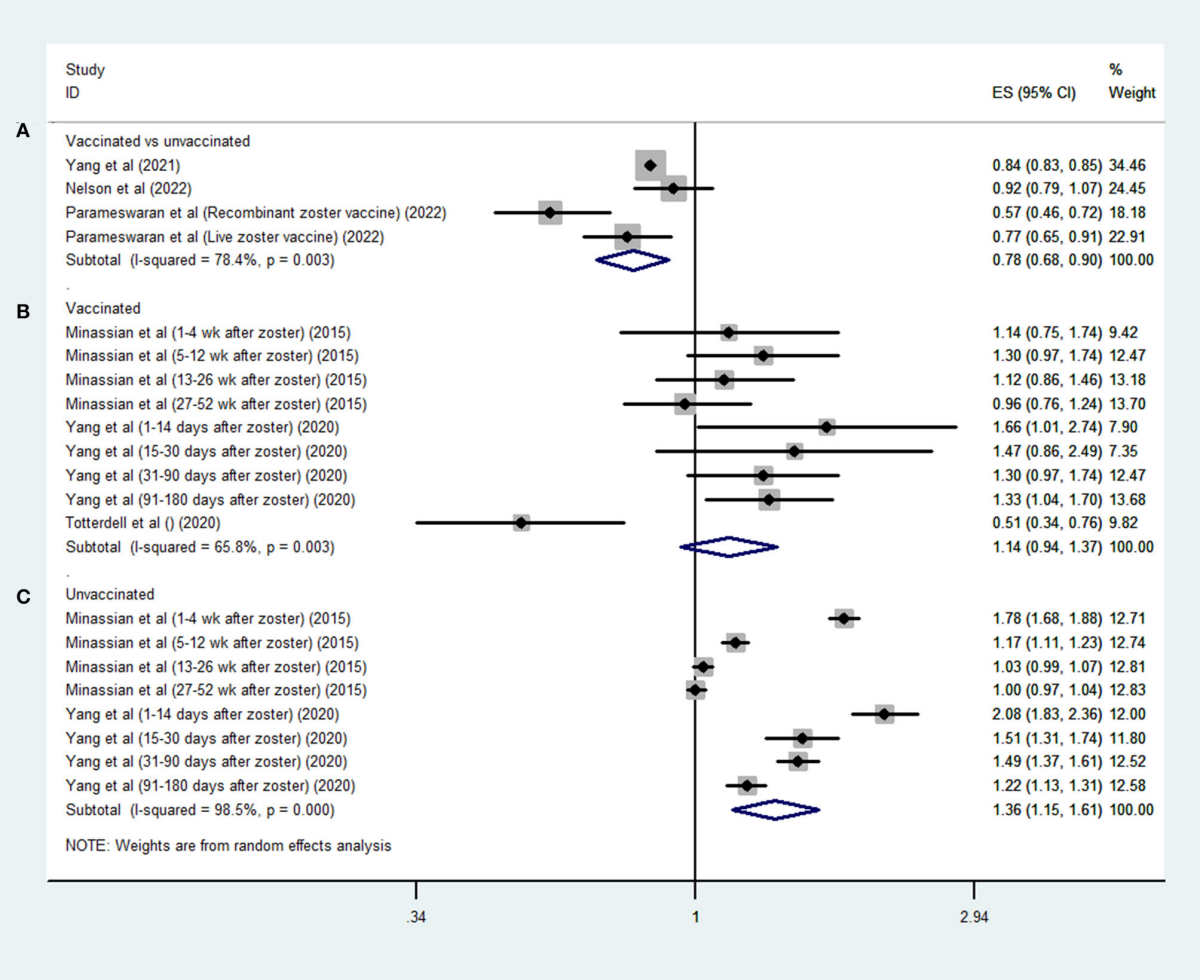
Forest plot of the overall risk of stroke in relation to HZV in **(A)** vaccinated vs. unvaccinated patients; **(B)** unvaccinated patients' own control; and **(C)** vaccinated patients' own control.

Three studies (17–19) evaluated the effect of HZV on stroke by comparing vaccinated and unvaccinated patients; the pooled data demonstrated that patients who received HZV were less likely to suffer from a stroke than those who did not (OR, 0.78; 95% CI 0.68–0.9; *I*^2^ = 78.4%; *P* = 0.001; Figure 2A).

Three SCCS studies (14–16) assessed the risk of stroke in defined periods after HZ compared with other periods. Among the vaccinated patients, there was no positive association between HZ infection and stroke risk (OR, 1.14; 95% CI 0.94–1.37; *I*^2^ = 65.8%; *P* = 0.181; Figure 2B); however, a higher risk of stroke was observed among unvaccinated patients after HZ infection (OR, 1.36; 95% CI 1.15–1.61; *I*^2^ = 98.5%; *P* < 0.001; Figure 2C).

### Characteristics of the included studies on the association between antiviral treatment and stroke

Table 2 presents the details of the seven studies that investigated the association between antiviral treatment and stroke risk; there were two SCCS studies (16, 23) and five cohort studies (20–22, 24, 25). The studies were published between 2010 and 2022. Three studies (16, 24, 25) were conducted in the United States, two (20, 21) in Asia, and two (22, 23) in Europe. Only one (25) was a hospital-based study; the others were population-based studies that analyzed database data. According to the NOS quality scores, six studies were deemed to be of high quality, with only one categorized as low quality. The score breakdown is presented in Supplementary Tables S3, S4.

**Table 2:** Characteristics of the included studies investigating the association between antiviral treatment and cerebrovascular events among HZ patients.

**References, study location**	**Study design**	**Study period**	**Age**	**Ascertainment of HZ**	**Comparison**	**Definition of cerebrovascular events**	**Ascertainment of antiviral treatment**	**Adjustment**	**Quality**
Lin et al. (20), Taiwan	Cohort	2003–2004	≥18	ICD-9-CM	Antiviral vs. No-antiviral	ICD-9-CM	Taiwan's National Health Insurance Research Database	No	7
Sreenivasan et al. (22), Denmark	Cohort	1995–2008	≥18	ICD-10	Antiviral vs. no-antiviral	ICD-10	Danish National Register of Medicinal Product Statistics	Age, gender, and calendar period	7
Langan et al. (23), UK	SCCS	1987–2012	≥18	ICD-10	Pre-antiviral vs. after-antiviral	ICD-10	Clinical Practice Research Datalink	Age	7
Calabrese et al. (24), USA	Cohort	2006–2013	≥65	ICD-9-CM	Antiviral vs. no-antiviral	ICD-9-CM	Medicare claims data	Age, gender, race, diabetes, hypertension, atrial fibrillation, transient ischemic attack, glucocorticoid use	9
Yang et al. (16), USA	SCCS	2008–2017	≥65	ICD-9-CM or ICD-10-CM	Pre-antiviral vs. after-antiviral	ICD-9-CM or ICD-10-CM	Medicare Provider Analysis and Review	FDR adjusted	8
Kim et al. (21), Korea	Cohort	2003–2014	≥18	ICD-10	Antiviral vs. no-antiviral	ICD-10	NHIS-NSC data	Gender, household income, medical history, steroid, antithrombotic, statin	9
Meyer et al. (25), USA	Cohort	2006–2016	≥65	Clinical charts	Antiviral vs. No-antiviral	Clinical charts	Clinical charts	No	5

### Meta-analysis of antiviral treatment and risk of stroke

**Figure 3:**
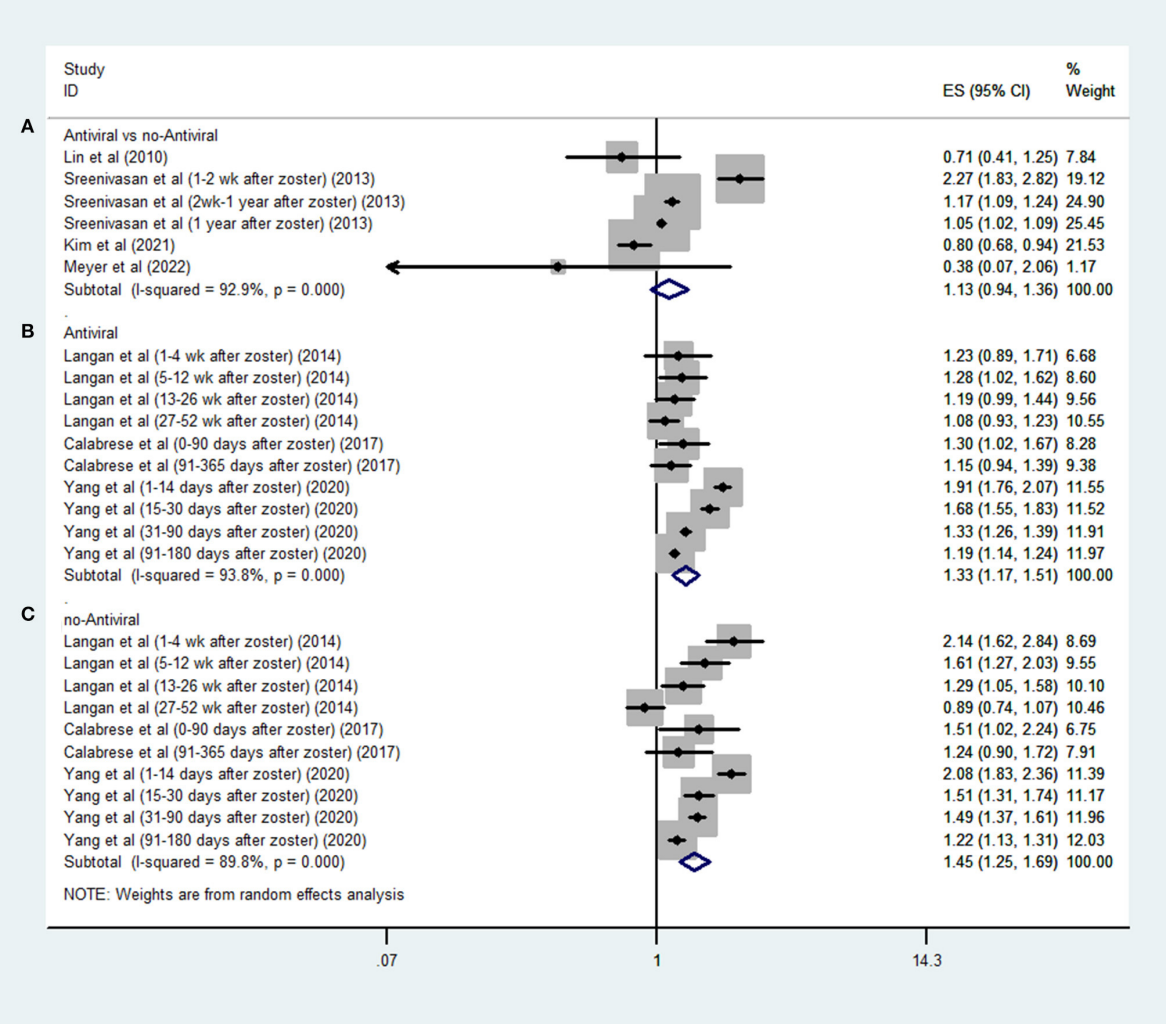
Forest plot of the overall risk of post-HZ stroke in relation to antiviral treatment (HZV): **(A)** antiviral vs. no-antiviral; **(B)** no-antiviral self-control; and **(C)** antiviral self-control of.

Four studies (20–22, 25) evaluated the effect of antiviral treatment on stroke by comparing treated and untreated patients with HZ; the pooled data demonstrated that antiviral treatment was not associated with a reduced risk of stroke in these patients after HZ (OR, 1.13; 95% CI 0.94–1.36; *I*^2^ = 92%; *P* = 0.201; Figure 3A).

Three SCCS studies (16, 23, 24) evaluated the effect of antiviral treatment on the risk of stroke in defined periods after HZ compared to other periods; the pooled data demonstrated that both treated (OR, 1.33; 95% CI 1.17–1.51; *I*^2^ = 93.8%; *P* < 0.001; Figure 3B) and untreated (OR, 1.45; 95% CI 1.25–1.69; *I*^2^ = 89.8%; *P* < 0.001; Figure 3C) patients were at a higher risk of stroke after HZ.

## Discussion

This systematic review and meta-analysis comprehensively investigated the current evidence on the effects of HZV and antiviral treatment on the risk of stroke; the pooled results showed that HZV was associated with a decreased risk of stroke, whereas no such protective modified effect was observed in patients with HZ who received antiviral treatment. The marked methodological variation among the studies and the limited number of included studies warrant a cautious interpretation of these findings.

Previous studies (10, 11, 26, 32) have focused on the relationship between HZ and stroke risk. Several biological mechanisms could explain such an association. One possible explanation is viral replication in the cerebral arteries. HZ virus can invade the arterial walls and thereby induce vasculopathy, which ultimately results in thrombosis, occlusion, infarction, aneurism, and hemorrhage (33). Inflammation also plays an important role in the etiology of stroke. A clinical study found inflammatory cells in the adventitia and intima in both early and late VZV vasculopathy (34). The inflammatory cytokines secreted by these inflammatory cells can potentially disrupt pre-existing atherosclerotic plaques (35). Consistent with the preclinical findings, the recent meta-analysis (10) based on 12 epidemiologic studies demonstrated an increased risk of stroke in the short term after herpes zoster infection. Therefore, prevention and treatment of HZ may modify the associated risk of stroke.

In previous studies, the live HZV vaccine was 51%−65% efficacious against zoster and post-herpetic neuralgia, and this increased to 90% efficacy for the recombinant zoster vaccine (12, 13). The protective effect of HZV was observed in our analysis by comparing the incidence of stroke between vaccinated and unvaccinated individuals. However, the pooled analysis of the SCCS studies did not support an effect of HZV in terms of modifying the risk of stroke. This may result from the designs of such studies. SCCS is a novel strategy to control for between-person confounders by comparing the risk and reference periods in each patient (36). Risk periods are defined as during or after an exposure. Considering the proven risk of stroke after HZ, the null association in vaccinated patients with HZ implies a protective effect of HZV. Meanwhile, the meta-analysis of the SCCS studies found an increased risk of stroke in unvaccinated patients with HZ.

Antiviral drugs are mainly used to treat acute HZ, and they can relieve pain, accelerate the healing of skin lesions, and reduce the spread of the virus (37). Theoretically, it follows that antiviral treatment may have the potential to reduce post-zoster stroke by reducing inflammation. However, our findings did not support the protective role of antiviral treatment on stroke, which may be due to the following reasons. First, antiviral treatment is likely to be used mainly in patients with severe symptoms. It is reasonable to speculate that a more severe disease confers a higher risk of stroke; further studies should consider the severity of HZ. Second, it is recommended that antiviral treatment should be started within 3 days of the onset of HZ. Early antiviral therapy has been proven to reduce the risk of post-herpetic neuralgia and other complications (38). Only one study included in our analysis evaluated the effect of the timing of antiviral treatment on the risk of cerebrovascular disease and found a protective role for prompt but not for delayed antiviral treatment (25). Therefore, delayed antiviral treatment may underestimate the beneficial effect of antiviral treatment on stroke. Third, patient age varied among the included studies, and increasing age is associated with a higher risk of stroke (39). Due to the low incidence of stroke in young people, the wide age range of the subjects enrolled in several of the included studies may have reduced our chance of finding a protective effect. Accordingly, one included study (21) performed a further age-stratified analysis and observed a protective effect of antiviral treatment only in patients aged ≥50 years. Hence, the protective effect of antiviral treatment may be age-dependent.

To the best of our knowledge, this study is the first systematic review of the effects of HZV and antiviral treatment on the risk of stroke. However, several limitations should be taken into account. First, the small number of included studies may have influenced the accuracy of our findings. Second, residual unknown confounders are always a concern in observational studies. Further well-designed studies that consider more covariates should examine these associations. Finally, the included studies were very heterogeneous in terms of data sources, definitions of stroke, age of enrolled subjects, and type of vaccination examined.

In conclusion, our findings suggest that HZV, but not antiviral treatment is associated with a reduced risk of post-HZ stroke. However, these conclusions should be interpreted with caution because of the high study heterogeneity and potential unknown confounders. Further well-designed studies with larger samples are needed to verify our findings.

## Data availability statement

The original contributions presented in the study are included in the article/Supplementary material, further inquiries can be directed to the corresponding author.

## Author contributions

A-jL and Y-hJ searched the library and drafted the manuscript. Y-bD and H-yJ extracted the data and revised all articles. A-jL designed the manuscript. All authors reviewed the manuscript.
