# Supplementary material for: Effects of herpes zoster vaccination and antiviral treatment on the risk of stroke: a systematic review and meta-analysis
Source: Front Neurol. 2023 May 17;14:1176920. doi: 10.3389/fneur.2023.1176920 (PMC10231675; doi:10.3389/fneur.2023.1176920)
Supplement: Supplementary file 1 [file Table_1.DOCX]

| **Table S1**  NOS for Assessment of Quality of Included Studies: Cohort Studies | | | | | | | | |
| --- | --- | --- | --- | --- | --- | --- | --- | --- |
| Study | Selection | | | | Comparability | | Outcomes | |
|  | Representativeness of exposed cohort? | Selection of the nonexposed cohort? | Ascertainment of exposure? | Demonstration that outcome of interest was not represent at the start of the study | Comparability of Cohort* | Assessment of outcome | Was follow-up long enough for outcomes to occur | Adequacy of follow up of cohorts |
| Yang et al, 2020, USA | ★ | ★ | ★ | ★ | ★★ | ★ | ★ | ★ |
| Nelson, 2022, USA | ★ | ★ | ★ | ★ | ★★ | ★ | ★ | ★ |
| Note: A star denotes a score of 1; * A maximum of 2 stars can be allotted in this category | | | | | | | | |

| **Table S2** NOS for Assessment of Quality of Included Studies: Case-Control Studies | | | | | | | | |
| --- | --- | --- | --- | --- | --- | --- | --- | --- |
| Study | Selection | | | | Comparability | Exposure | | |
|  | Is the case definition adequate | Representativeness of cases | Selection of controls | Definition of controls | Study controls for at least 3 additional factors | Ascertainment of exposure | Same method of ascertainment of exposure | Nonresponse rate |
| Minassian et al, 2015 | ★ | **★** | ★ | ★ | ★★ | **★** | ★ | ★ |
| Totterdell et al, 2020 | — | **★** | ★ | ★ | ★★ | **★** | ★ | ★ |
| Yang et al, 2020 | ★ | **★** | ★ | ★ | ★★ | **★** | ★ | **★** |
| Parameswaran, 2022 | ★ | **★** | ★ | ★ | ★★ | **★** | ★ | — |

| **Table S3**  NOS for Assessment of Quality of Included Studies: Cohort Studies | | | | | | | | |
| --- | --- | --- | --- | --- | --- | --- | --- | --- |
| Study | Selection | | | | Comparability | | Outcomes | |
|  | Representativeness of exposed cohort? | Selection of the nonexposed cohort? | Ascertainment of exposure? | Demonstration that outcome of interest was not represent at the start of the study | Comparability of Cohort* | Assessment of outcome | Was follow-up long enough for outcomes to occur | Adequacy of follow up of cohorts |
| Lin et al, 2010 | ★ | ★ | ★ | ★ | — | ★ | ★ | ★ |
| Sreenivasan et al, 2013 | ★ | ★ | ★ | ★ | — | ★ | ★ | ★ |
| Calabrese et al, 2017 | ★ | ★ | ★ | ★ | ★★ | ★ | ★ | ★ |
| Kim et al, 2021 | ★ | ★ | ★ | ★ | ★★ | ★ | ★ | ★ |
| Meyer et al, 2022 | — | ★ | ★ | ★ | — | — | ★ | ★ |
|  | | | | | | | | |

| **Table S4** NOS for Assessment of Quality of Included Studies: Case-Control Studies | | | | | | | | |
| --- | --- | --- | --- | --- | --- | --- | --- | --- |
| Study | Selection | | | | Comparability | Exposure | | |
|  | Is the case definition adequate | Representativeness of cases | Selection of controls | Definition of controls | Study controls for at least 3 additional factors | Ascertainment of exposure | Same method of ascertainment of exposure | Nonresponse rate |
| Langan et al, 2014 | ★ | **★** | ★ | ★ | ★ | **★** | ★ | — |
| Yang et al, 2020 | ★ | **★** | ★ | ★ | ★★ | **★** | ★ | — |
